# Lithium and Water: Why Hydrological Limits Will Define a Just Energy Transition

**DOI:** 10.1002/gch2.70151

**Published:** 2026-08-30

**Authors:** Ricardo O. Barra, Fernanda Álvarez‐Amado, Víctor Parra, Marcela Salgado, Susana Alvarez, Amaya Alvez, Leopoldo Gutiérrez

**Affiliations:** ^1^ Departamento De Sistemas Acuáticos Facultad De Ciencias Ambientales y Centro EULA Chile Universidad De Concepción Barrio Universitario S/N Concepción Concepcion Chile; ^2^ Departamento De Ciencias De La Tierra Facultad De Ciencias Químicas Universidad De Concepción Barrio Universitario S/N Concepción Concepcion Chile; ^3^ Departamento De Ingeniería Ambiental Facultad De Ciencias Ambientales y Centro EULA Universidad De Concepción Barrio Universitario S/N Concepción Concepcion Chile; ^4^ Departamento De Planificación Territorial y Sistemas Urbanos Facultad De Ciencias Ambientales y Centro EULA Universidad De Concepción Barrio Universitario S/N Concepción Concepcion Chile; ^5^ Departamento De Derecho Público y de Historia y Filosofía Del Derecho Facultad De Ciencias Jurídicas y Sociales Universidad De Concepción Barrio Universitario S/N Concepción Concepcion Chile; ^6^ Departamento De Ingeniería Metalúrgica Facultad De Ingeniería Universidad De Concepción Barrio Universitario S/N Concepción Concepcion Chile

**Keywords:** Chile, Environmental justice, hydrological limits, lithium extraction, Salar de Atacama, socioecological transition, water governance

## Abstract

Lithium is widely portrayed as a cornerstone of the global energy transition. Yet prevailing narratives conceptualize lithium primarily as a mineral resource while treating water impacts as secondary externalities. This Perspective argues that in arid and hyper‐arid salt‐flat systems, lithium extraction is fundamentally constrained by hydrological boundaries rather than by mineral availability or technological efficiency. Using the Salar de Atacama in northern Chile as an emblematic global case, we contend that brine‐based lithium extraction constitutes a large‐scale intervention in coupled hydrosocial systems, directly implicating a boundary for freshwater. We challenge techno‐optimistic expectations surrounding efficiency improvements and emerging technologies such as direct lithium extraction, emphasizing instead cumulative impacts, persistent uncertainty, and uneven distributions of risk and benefit. Building on the water boundaries framework, we propose a shift toward water‐bounded lithium governance grounded in basin‐scale extraction limits, precaution under uncertainty, legal recognition of brines as water, and meaningful indigenous participation linked to extraction thresholds. Without re‐centering water as the defining constraint, the energy transition risks reproducing environmental degradation and social injustice under the guise of sustainability.

## Graphical abstract

Lithium extraction is reframed as a water governance challenge rather than solely a mining issue. Water‐Bounded Lithium Governance places hydrological limits at the center of decision‐making, offering a new pathway to reconcile critical mineral supply with environmental integrity, indigenous rights, and a just energy transition.

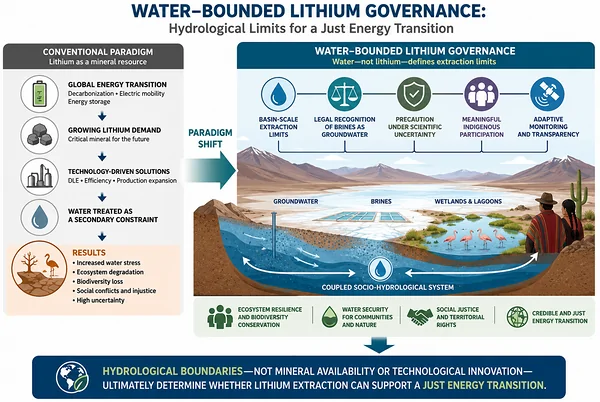

## 1 Introduction

Lithium has emerged as a strategic material at the center of the global energy transition, driven by accelerating demand for electric vehicles, grid‐scale energy storage, and low‐carbon technologies [1]. Policy and industry discourse commonly portray the expansion of lithium extraction as both necessary and broadly compatible with sustainability objectives [2]. However, this dominant framing rests on a critical assumption: that lithium extraction can be decoupled from fundamental biophysical limits [3]. Here, we understand these limits as the hydrological, ecological, and geochemical thresholds—basin‐scale water and brine budgets, ecosystem water requirements, and aquifer recharge rates—beyond which continued extraction compromises the long‐term integrity of the coupled hydrosocial system. Environmental concerns are typically addressed through promises of technological innovation, improved efficiency, alternative extraction technologies such as direct lithium extraction (DLE), and voluntary environmental standards (e.g., certification schemes such as the Initiative for Responsible Mining Assurance or ISO 14001 environmental management standards).

This Perspective challenges that assumption. We argue that prevailing mineral‐centered approaches to lithium governance tend to obscure the central role of water as the primary limiting factor shaping both the environmental sustainability and the social legitimacy of lithium extraction, particularly in arid regions [4]. In brine‐based operations such as the Salar de Atacama, lithium production is inseparable from large‐scale interventions in coupled groundwater and brine systems that sustain fragile ecosystems, endemic biodiversity, and human livelihoods [1, 5].

Despite growing recognition of socio‐environmental impacts associated with lithium extraction, governance frameworks remain poorly aligned with hydrological realities [4, 6]. In major brine‐producing regions, brines are commonly regulated as mineral resources rather than as integral components of basin‐scale water systems, often allowing extraction to expand in the absence of enforceable water budgets or ecological thresholds. Technological solutions, including DLE, are increasingly promoted as pathways to sustainability [7], yet often without adequately addressing system‐wide water requirements, cumulative impacts, or the risks associated with persistent uncertainty regarding coupled freshwater–brine systems. This is precisely the governance gap that technology‐centered narratives, discussed next, frequently fail to close: technological fixes are proposed without addressing the prior and more fundamental absence of enforceable water budgets. Existing governance frameworks remain largely mineral‐centered. Lithium‐bearing brines are generally regulated as mineral deposits despite functioning as integral components of basin‐scale hydrological systems. This regulatory perspective tends to separate mineral extraction from water governance, limiting the capacity to assess cumulative hydrological impacts and ecosystem resilience.

Using the Salar de Atacama as an emblematic global case rather than a local exception, this Perspective advances three core arguments. First, lithium brines must be governed as components of coupled hydrological systems rather than solely as mineral resources [4]. Second, hydrological limits—not technological capacity—define the ultimate boundary conditions for sustainable lithium extraction [9]. Third, achieving a just energy transition requires governance frameworks that integrate basin‐scale hydrological thresholds, precaution under uncertainty, transparent monitoring, and meaningful Indigenous participation [6]. Together, these arguments call for a rethinking of lithium governance within global sustainability transitions. Together, these principles define what we term water‐bounded lithium governance.

## 2 Lithium in Chile

**FIGURE 1:**
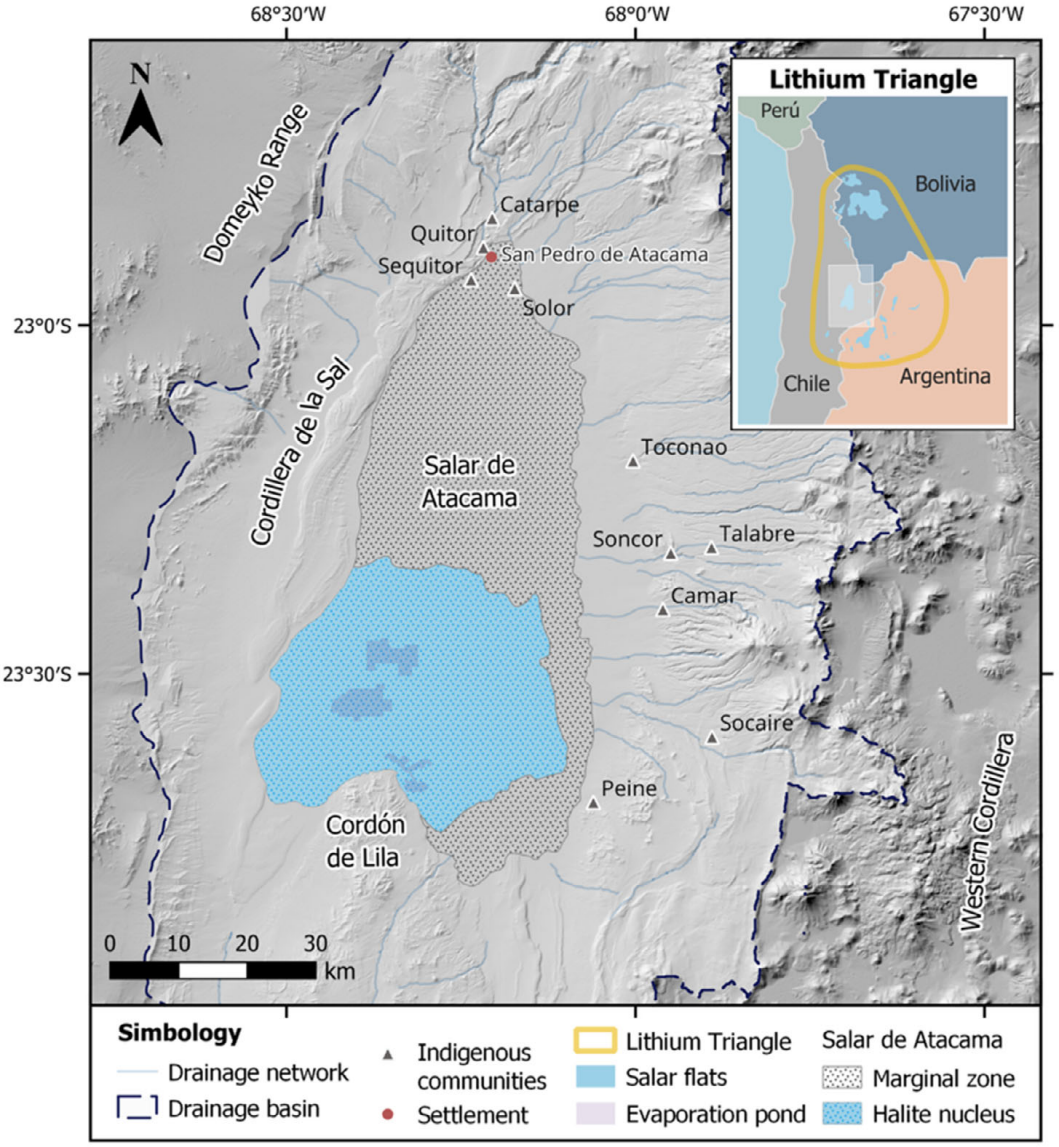
The Salar de Atacama within the Lithium Triangle of South America. The figure shows the extent of the Salar de Atacama basin, the halite nucleus and evaporation pond areas associated with lithium production (SQM and Albemarle), as well as the distribution of nearby Indigenous communities. Together, these elements highlight the spatial overlap of lithium extraction, water resources, and local communities within a shared socio‐hydrological system.

Lithium production in Chile is concentrated in the Salar de Atacama (Figure 1), a high‐altitude basin within one of the driest deserts on Earth. Hydrologically, the basin exhibits high solar radiation, extreme evaporation, very low precipitation, and a fragile water balance supported by complex groundwater–surface water interactions [4, 10, 11, 12]. Recent hydrological evidence suggests that a substantial fraction of freshwater inflows to the Salar de Atacama is sustained by groundwater with relatively long residence times, reflecting recharge that predates the modern climatic regime and challenging assumptions of steady‐state water availability [10]. This indicates that freshwater recharge could occur over timescales that are considerably longer than those associated with lithium extraction, limiting the capacity of the system to recover from sustained groundwater and brine withdrawals. These hydrogeological characteristics highlight the importance of considering hydrological limits when evaluating the long‐term sustainability of lithium extraction. Industrial practice pumps Li‐rich brines from deep saline aquifers to large evaporation ponds, where water is progressively lost to the atmosphere, concentrating lithium‐bearing salts [1, 2]. Although solar evaporation reduces energy requirements relative to thermal processes, its dependence on intense evaporative losses under hyper‐arid conditions raises critical questions about long‐term hydrological sustainability, ecological impacts and resilience [2, 4].

Evidence from hydrological modeling and field observations indicates that brine extraction can perturb groundwater dynamics and influence freshwater–saline gradients at different parts of the basin [4]. These changes are difficult to monitor and attribute because the aquifer is heterogeneous, data access has historically been limited, and multiple stressors operate concurrently [11, 13, 14]. At the same time, analyses based on surface indicators have reported no significant reduction in lagoon surface areas over specific periods [15]. Rather than indicating the absence of impacts, this mixed evidence highlights the need for integrated basin‐scale monitoring, combining hydraulic heads, fluxes and chemistry in aquifers, and ecological indicators to better understand cumulative effects and to design adaptive governance.

The socio‐hydrological context is equally critical. The Salar de Atacama is not only a lithium‐producing basin but also an ancestral territory inhabited by Atacameño (Lickanantay people in the kunza language) communities that have coexisted with lagoons, wetlands, and salars for centuries and have developed local forms of territorial and water governance [16]. Contemporary livelihoods combine agriculture and livestock with wage labor in lithium and copper mining and tourism. Competing water demands from copper mining, local settlements, as well as tourism create additional pressures and complicated governance. In April 2023, the National Lithium Strategy signaled an expansion of production and the adoption of new technologies, alongside commitments to mitigate socio‐environmental impacts [17].

A clear distinction between lithium's status as a non‐concessible mineral and the legal nature of brine is essential for a robust legal analysis of salt flat governance. The “non‐concessible” status, established in 1979 and ratified by the 1983 Mining Code, is a matter of ownership and entitlement; it dictates that lithium is reserved for the State and can only be exploited directly by it, its companies, or through Special Lithium Operation Contracts (CEOLs). This classification addresses who has the right to extract the mineral but does not define the medium in which it is found. There are numerous bills currently in transition (such as Bulletins 16785‐07 and 16117‐08) that seek to: Redefine lithium's strategic value; eliminate its non‐concessible status and restrict or suspend private contracts and administrative concessions.

## 3 Water and Boundary Interactions Relevant to Brine‐Based Lithium

Brine‐based lithium intersects three boundaries most clearly: freshwater use, biosphere integrity and novel entities [18, 19]—the last referring, within the planetary boundaries framework, to chemicals, materials and organisms newly introduced into the Earth system whose effects are not yet well characterized. In high‐Andean salt‐flat ecosystems, groundwater regimes sustain wetlands and endemic species. Perturbations arising from cumulative withdrawals and evaporation‐driven losses may reduce ecological water availability, alter salinity gradients and degrade habitat quality for iconic species such as flamingos [20, 21] as well as for benthic macroinvertebrate communities, halophytic and hydrophytic vegetation, and the microbial mats that underpin primary production in these systems. These pressures are amplified by the basin's low recharge and high evaporative demand [8, 10].

Novel entities encompass process chemicals and airborne particulates generated by mining activities. Their fate and effects in saline soils and waters remain uncertain and context‐dependent; they warrant monitoring but should not be overgeneralized beyond the evidence [2, 22]. Because the fate, transport and cumulative effects of these substances in saline aquifers remain difficult to quantify with current data, they cannot yet serve as a reliable basis for enforceable governance thresholds; water, by contrast, can be measured, modeled and capped at the basin scale, which is why we treat it as the primary and most tractable governance boundary, while treating novel entities as an important but secondary object of precautionary monitoring. Emphasizing water as a boundary resource focuses decisions on variables that can be measured and managed at basin scale: aquifer status (levels and fluxes), total withdrawals by sector, ecological dependencies and thresholds, and the distribution of risks among communities and ecosystems [6].

The “legal nature of brine” is a regulatory and environmental question regarding the fluid itself and is the legal debate we would focus on in this paper in the following sections. While brine is currently treated under a mineral logic as part of a “deposit,” we argue it is functionally groundwater integrated into complex hydrological systems. The legal framework defines a “salar” as a saline deposit in a closed basin containing occluded brines, focusing strictly on its mineral potential. The legal interpretation of lithium brine is a grey zone in Chile; it is currently regulated as a mineral resource rather than continental water [23]. If brine were conceived as groundwater under the 2022 Water Code, its extraction would be subject to integrated basin management, the prioritization of human consumption, and the protection of the hydrogeological balance. This legal rigidity about the legal nature of brine contrasts with the scientific reality that brine is fundamentally groundwater. Integrating this hydrogeological perspective is essential to move from an “exclusively mineral” logic toward a sustainable model that prioritizes human consumption and ecosystem health. In our viewpoint, the legal regulation of lithium brine is a non‐neutral crossroads that determines how economic exploitation is reconciled with the preservation of fragile ecosystems. One ongoing debate is whether the public interests regarding environmental damage to aquifers are subordinated to the fiscal benefits of Lithium mining.

In this perspective, we define water‐bounded lithium governance as a governance framework in which basin‐scale hydrological resilience establishes the upper boundary within which technological innovation, mineral production, economic development, and social agreements may occur. Under this framework, extraction thresholds are determined by ecosystem functioning rather than production targets.

## 4 Fair Socioecological Transition in Chile

Socio‐ecological transitions are understood as processes of profound and structural change in the relationship between society and nature. In this sense, Andrade y Corzo [24] define them as processes aimed at shaping a new culture and a society where demands do not exceed supply or the limits of nature but rather contribute from all disciplines to its regeneration and health.

Chilean policy frames a fair socioecological transition around social and environmental justice, citizen participation, social dialogue, ecosystem protection, decent work, gender equality and human rights [25]. In the Atacama salt flats, this agenda is tested by the coexistence of expanding extractive projects, water scarcity and territorial claims from Indigenous organizations [6, 26]. To lithium governance, three operational components are most relevant:

- Recognition of hydrological complexity: brines and freshwaters are interconnected; decisions should treat the basin as a coupled socio‐ecological system [8, 11, 14].
- Meaningful participation: Indigenous organizations must be involved in basin‐level decisions, including quota‐setting, monitoring design and benefit‐sharing [6, 26].
- Transparency: cumulative‐impact information, models and monitoring data should be publicly accessible to enable scrutiny and adaptive management [27].

These components align with international commitments to free, prior and informed consultation and with domestic aspirations for equitable development. They also create enabling conditions for more credible policy, reduce conflict and support learning in a context of uncertainty [28]. Finally, it is necessary to examine whether the current National Lithium Strategy truly aligns with the Paris Agreement's goals of achieving a climate‐resilient and decarbonized economy by 2050.

## 5 Local Indigenous Communities and Hydrosocial Dynamics

The Atacameño people are organized into various communities within the Atacama Salt Flat basin, primarily in the San Pedro de Atacama commune, where 62.5% of inhabitants identify as indigenous. They employ transhumance strategies across diverse ecological levels, while the State has cataloged 2.3 million hectares as traditional occupation lands; tensions remain because indigenous ancestral rights extend from mountain springs to irrigated oases. The Chilean State initially sought to limit recognition to fixed settlements (towns and villages) and formal official titles provided by the State, international standards and indigenous demands have pushed for criteria that respect mobility, traditional use, and the spiritual connection to the entire hydro‐social system of salt flats.

The state's “permanent settlement” criterion often fails to recognize these territories, as communities traditionally move between micro‐territories depending on the season. This recent expansion of lithium mining has generated constant tension between the State's economic valuation and the communities' territorial‐cultural valuation. National courts have been the primary forum for disputes involving public law, indigenous rights and water management. Community relations are currently regulated by special contracts (CEOLs) and subsequent arbitral modifications. A 2021 Supreme Court ruling mandated that the State conduct indigenous consultations, following human rights standards, for new lithium contract CEOLs (SC ruling rol N° 99–2022). In 2023, negotiations with SQM included prior, free, and informed consultations according to ILO Convention 169. While these processes resulted in significant financial contributions for community projects—potentially reaching US$10‐15 million plus a percentage of sales by 2030—they are not legally binding agreements. According to ethnographic and documentary research on Atacameño organizations following these agreements [26], for some community leaders and organizations these consultations function more as negotiation tools to reduce asymmetry with mining companies than as mechanisms to protect environmental interests or indigenous rights, while other organizations have endorsed the agreements as a source of community investment. In contrast, high‐stakes commercial conflicts related to the financial terms of the exploitation contracts were solved in 2024 through arbitration by the International Court of Arbitration of the International Chamber of Commerce (ICC). Community relations are currently regulated by these special contracts (CEOLs). Based on their arbitral modifications over time, this has direct impact on how the companies interact with the inhabitants of the territory.

Communities and organizations have taken divergent positions: some, such as those engaging through the council of atacameño people (CPA's) negotiated agreements, have negotiated direct payments, environmental monitoring schemes and benefit‐sharing agreements with companies; while others—including community members interviewed and organizations documented by Lorca et al. [26, 38]—have opposed expansion projects, citing hydrosocial impacts, loss of ancestral water rights, and deficits in basic services. This divergence reflects both differentiated exposure to mining impacts across communities and internal disagreement about whether negotiation or opposition better protects territorial rights [6, 26, 30, 31].

We note that “Atacameño” (Lickanantay) identity is itself a historically constructed and internally differentiated category; questions of who is recognized as an Indigenous community and who speaks on behalf of “the Atacameño people” have been contested and have shifted over successive waves of state recognition and organizational reconfiguration [38].

Communities such as Peine (∼600 people) and Toconao (∼800) and smaller settlements (Camar, Socaire, Talabre) maintain agriculture and livestock practices adapted to high‐Andean conditions, including cultivation in ravines and small‐scale husbandry of camelids and small ruminants [28, 29]. Over recent decades, wage employment in lithium and copper mining has grown. Some communities have negotiated direct payments, environmental monitoring schemes and benefit‐sharing agreements with companies; others have opposed expansion projects, citing hydrosocial impacts and deficits in basic services. Published studies demonstrate that Indigenous organizations within the Salar de Atacama express diverse positions regarding lithium extraction. While some communities emphasize employment opportunities and local investment, others prioritize hydrological protection, cultural heritage, and territorial autonomy. These differences reinforce the need for governance arrangements capable of accommodating plural values rather than assuming a single Indigenous position [6, 26, 30, 31].

Legal treatment of brines as a mineral rather than as water complicates governance because it obscures ecological roles and Indigenous knowledge systems about the hydrology of salt flats [6]. A fair‐transition approach requires acknowledging divergent local preferences—some residents may welcome employment and investment while others prioritize ecological integrity and cultural heritage—and ensuring free, prior and informed consultation embedded in decisions about quotas, monitoring and the distribution of revenues [22, 30]. Finally, Hickel and Kallis [3] question the premise of the absolute decoupling between economic growth and ecological sustainability, arguing that transitions that do not challenge the logic of growth are structurally insufficient to respond to the ongoing planetary crisis.

## 6 Environmental Impacts: Hydrology, Biodiversity and Process Trade‐Offs

The impact on water resources represents the greatest environmental threat, as evaporation‐based extraction consumes critical volumes of water in extremely arid zones. Currently, 89% of water extraction in the Atacama Salt Flat basin is allocated to the mining sector. Consequently, the General Water Directorate (DGA) has declared certain rivers exhausted and prohibited further groundwater extraction. Despite this, a projected exponential increase in mining activity persists. Chile's National Lithium Strategy explicitly seeks to expand lithium production and develop new projects beyond current operations [17]. In the Salar de Atacama, the Codelco‐SQM agreement targets an additional 300 000 tonnes of lithium carbonate equivalent (LCE) during 2025–2030 and annual production of 280 000–300 000 tonnes from 2031 onward [39]. In parallel, ENAMI and Rio Tinto are advancing the Salares Altoandinos project, supported by an estimated investment of US$3 billion [39]. These examples clarify the basis for anticipating substantial expansion and reinforce the need to assess future production against basin‐scale hydrological limits.

Brine‐based mining depends on groundwater. Large‐volume pumping to evaporation ponds includes long‐term depletion of groundwater storage, with limited natural recovery due to extremely low recharge rates in hyper‐arid settings [1, 2]. Potential hydrological impacts include depletion of aquifers that support meadows, wetlands and lakes; altered freshwater–saline gradients; and risks to species such as flamingos that rely on specific hydrological and ecological conditions [20, 21]. For hard‐rock operations, high water requirements for crushing, separation and refining also create pressures where water is scarce [32].

Technology choices have non‐trivial trade‐offs. Direct lithium extraction (DLE) could reduce reliance on extensive evaporation ponds and shorten residence times; however, several studies indicate that many DLE configurations may require additional freshwater and energy inputs [2], as well as generate waste streams that must be carefully managed. Context‐specific evaluation with full accounting of water consumption, energy, reagents and waste is therefore essential; claims of blanket superiority or inferiority are not supported by the heterogeneous evidence [2, 22]. Conventional evaporation relies primarily on solar energy and therefore has relatively low operational energy requirements, but it irreversibly removes large volumes of brine water through evaporation ponds. By contrast, reported freshwater requirements for DLE range widely, with some configurations exceeding 500 m^3^ per tonne of Li_2_CO_3_, more than ten times the freshwater demand reported for current evaporation‐based operations‐whereas the commercial Hombre Muerto facility reports approximately 71 m^3^ per tonne [2]. In conventional evaporation‐pond processing, approximately 95% of the water contained in the extracted brine is ultimately lost to the atmosphere [5]. Thus, although DLE can substantially reduce evaporative brine losses, its freshwater advantage depends strongly on washing requirements, water recycling, and brine reinjection. Energy trade‐offs are also substantial. A modeled conventional evaporation‐based route at Salar de Atacama requires approximately 6.2 kWh kg^−1^ Li_2_CO_3_ [39]. In comparison, the Clayton Valley DLE case requires approximately 59 kWh kg^−1^ Li_2_CO_3_ of combined heat and electricity, nearly one order of magnitude higher [22, 39]. DLE also reduces evaporation‐pond area and processing time, but may shift environmental burdens toward electricity, reagents, spent sorbents, and concentrated liquid‐waste streams [2, 22]. Therefore, Compatibility with the proposed framework should first be evaluated through net freshwater demand, brine extraction‐reinjection balance, changes in groundwater levels, fluxes and salinity, and compliance with basin‐scale ecological thresholds. Additional criteria include energy and carbon intensity, reagent consumption and toxicity, waste volume and hazardousness, and potential alteration of brine chemistry or aquifer properties [2, 22]. A technology would be considered compatible only if its cumulative impacts remain within enforceable hydrological limits, do not transfer environmental burdens to other media or communities, and are supported by transparent monitoring and adaptive management.

## 7 Water Use in the Salar de Atacama

**FIGURE 2:**
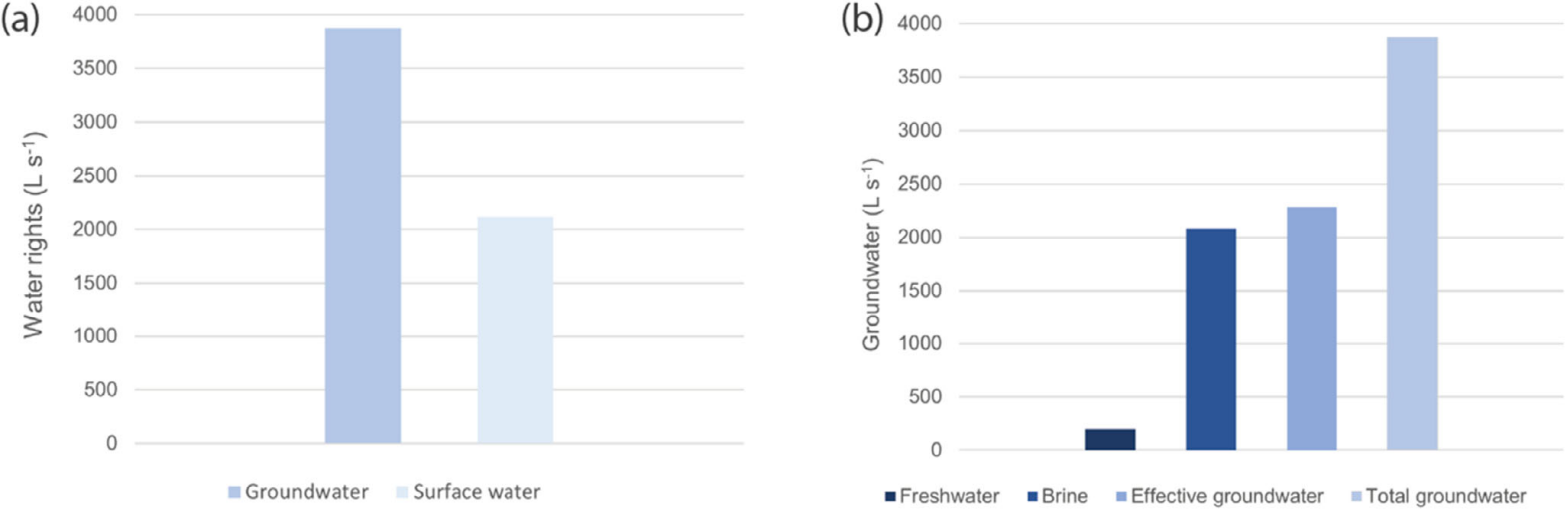
Surface and groundwater rights granted in the Salar de Atacama basin (a) and groundwater extraction with environmental approval granted to mining companies (b).

Water rights granted by the Dirección General de Aguas (DGA) differentiate surface water, which predominantly serves local communities, from groundwater, which is primarily used by mining and includes both freshwater withdrawals (e.g., copper) and brine pumping (lithium) [4, 11]. Figure 2a summarizes granted rights (surface ∼2115 L/s; groundwater ∼3873 L/s), while Figure 2b presents approved mean annual extraction flow rates [6]. Reported combined extractions have lowered water tables across parts of the basin, with implications for lakes and wetlands that rely on groundwater. Recharge from precipitation and runoff is insufficient to offset natural and human‐driven losses, raising concerns regarding long‐term hydrological sustainability [10, 11, 32].

The Lickanantay people, inhabiting the Salar de Atacama for 11 000 years, view water as a sacred, structural element of their ancestral territory. They demand that brine be legally recognized as water to protect fragile aquifers and cultural heritage. Indigenous claims emphasize territorial rights spanning from high‐mountain springs to irrigated oases, rejecting extraction models that ignore their ancestral knowledge. Consequently, they seek meaningful participation in basin‐level decision‐making, including quota‐setting and monitoring—grounded in international human rights standards and the principles of free, prior, and informed consent to prevent social injustice.

Recent hydrological evidence further challenges assumptions of steady‐state water availability in the Salar de Atacama, suggesting that a substantial fraction of freshwater inflows may be sustained by groundwater recharged under past climatic conditions rather than solely by modern precipitation [10, 33]. These findings underscore the importance of understanding groundwater residence times and recharge dynamics when evaluating the sustainability of lithium extraction in arid basins. If water availability responds over timescales that do not align with industrial extraction, hydrological limits may ultimately define whether lithium production can remain compatible with a just energy transition. However, the interpretation of impacts requires caution. Guzmán et al. [15] found no significant reduction in lagoon surface areas over the period analyzed, despite increased brine extraction, highlighting the limitations of surface indicators and the importance of integrating groundwater measurements and ecological data. At the same time, evidence of localized ground deformation associated with brine extraction suggests that some system responses may not be immediately visible, reinforcing the need for caution when interpreting hydrological impacts through single indicators alone [34]. Multiple stressors co‐occur: climate variability and longer‐term change, tourism‐related water use, and copper mining withdrawals upgradient of the salar [13, 14]. Basin‐scale assessments should recognize and quantify the cumulative and interacting drivers ‐including brine and freshwater extractions‐ and avoid attributing observed impacts to a single factor without sufficient evidence.

Actionable implications follow from this analysis. First, set basin‐scale extraction ceilings anchored in modeled aquifer status and ecological thresholds—for example, prioritizing ecological needs in wetlands supporting flamingo populations and other endemic biota [11, 21]. Second, reduce overall water use toward a sustainable range (e.g., hundreds of L/s rather than thousands) while ensuring community needs are met, and basic services are strengthened [11, 36]. Third, implement integrated monitoring that couples field measurements of water table with numerical modelling and chemistry with ecological indicators; align sensor networks and campaigns across companies and public agencies; and make data and models publicly accessible [4, 11]. Finally, evaluate process changes, including DLE and recycling initiatives, with full accounting of water, energy and waste trade‐offs; avoid technology lock‐in without robust evidence [2, 22, 37].

## 8 Outlook: Actors, Enabling Conditions and Near‐Term Levers

The rapid expansion of lithium extraction highlights a central tension within the global energy transition: the prioritization of supply growth over biophysical limits and social equity. As demand accelerates, governance approaches that treat lithium primarily as a mineral commodity while marginalizing water scarcity will increasingly collide with hydrological realities, particularly in arid and semi‐arid regions.

**FIGURE 3:**
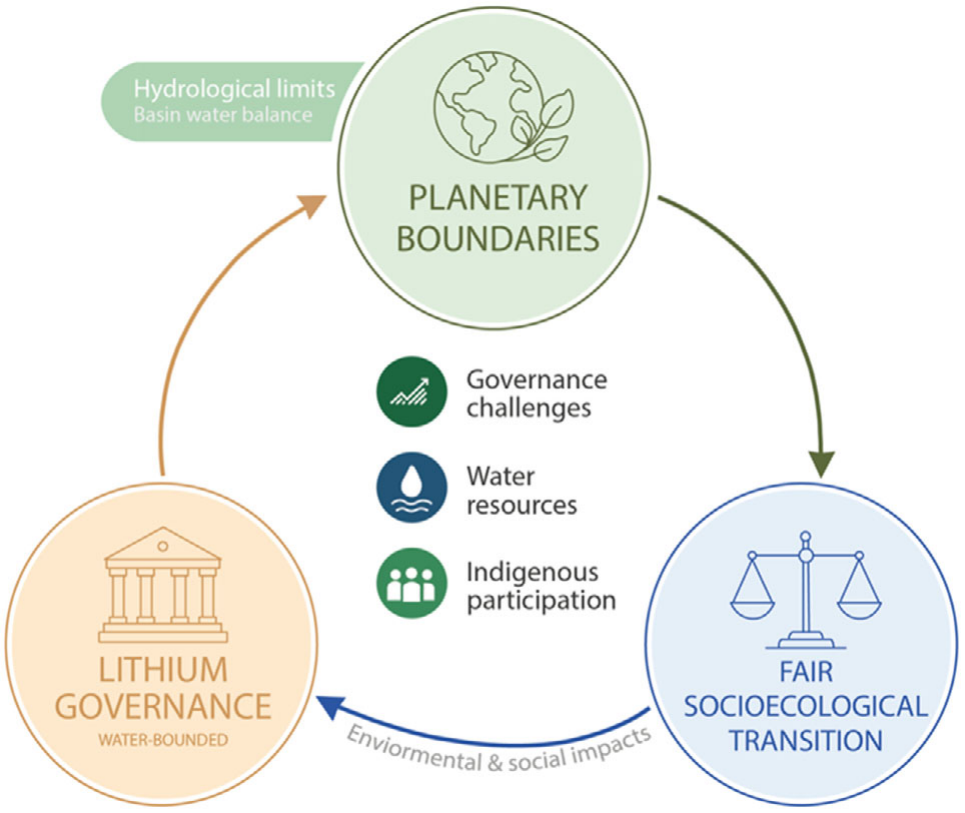
Conceptual integration of Planetary Boundaries, Fair Socioecological Transition, and Lithium Governance.

Re‐centering water as the defining constraint of lithium extraction requires moving beyond technological optimism. Efficiency gains and new extraction technologies may alter the form of environmental impacts, but they cannot eliminate the fundamental dependence of lithium production on finite and vulnerable water systems. Without enforceable basin‐scale extraction limits, efficiency improvements risk accelerating total withdrawals and intensifying cumulative impacts (Figure 3)

A water‐bounded approach to lithium governance demands several urgent shifts. Brines must be legally recognized as components of coupled water systems subject to enforceable hydrological caps. Scientific uncertainty should trigger precaution, adaptive management, and extraction ceilings rather than justify continued expansion. Indigenous participation must extend beyond procedural consultation toward substantive influence over extraction thresholds, monitoring, and long‐term basin futures.

Lithium brine in Chile faces a profound structural legal challenge regarding its dual nature as both a mineral and a water resource. Currently, the 1983 Mining Code treats brine exclusively as a mineral substance, enabling extraction through special state contracts. However, this mineral‐centered focus creates a fragmented regulatory framework that fails to account for brine's role in coupled hydrological systems. By neglecting the 2022 Water Code, this interpretation risks ecosystem degradation and the violation of Lickanantay indigenous rights. Legally resolving this classification is essential to prioritize human consumption and environmental sustainability within these fragile, hyper‐arid territories for all future generations.

Without such reorientation, lithium risks becoming another extractive pillar of sustainability discourse that externalizes ecological degradation and social costs to peripheral regions. Addressing climate change requires profound transformation of energy systems, but it does not suspend biophysical limits. A credible and just energy transition must be confronted—rather than bypass—the hydrological boundaries that ultimately govern life in arid landscapes.

## Funding

Instituto Milenio SECOS ANID ICN 2019‐015, ANID/ANILLO/ATE240013, ANID CTI CTI250001, Consorcio Tecnologico del Agua CoTH2O and the EULA Chile Center at the University of Concepción, Chile.

## Conflicts of Interest

The authors declare no conflicts of interest.

## Data Availability

The data that support the findings of this study are available from the corresponding author upon reasonable request.
